# Supplementary material for: The critical role of ultra-low-energy vibrations in the relaxation dynamics of molecular qubits
Source: Nat Commun. 2023 Mar 24;14:1653. doi: 10.1038/s41467-023-36852-y (PMC10039010; doi:10.1038/s41467-023-36852-y)
Supplement: Supplementary file 1 — Supplementary Information [file 41467_2023_36852_MOESM1_ESM.pdf]

Supplementary Information

The critical role of ultra-low energy vibrations in the relaxation dynamics of molecular qubits

E. Garlatti, A. Albino, S. Chicco, V.H.A. Nguyen, F. Santanni, L. Paolasini, C. Mazzoli, R. Caciuffo, F. Totti, P. Santini, R. Sessoli*, A. Lunghi* and S. Carretta*

*Corresponding authors. Email: lunghia@tcd.ie, roberta.sessoli@unfi.it, stefano.carretta@unipr.it

Supplementary Note 1 – Crystallographic Information

The Vanadyl tetraphenylporphyrinate system [VO(TPP)] crystallizes in a tetragonal structure (space group I4), with the V═O bond lying along the four-fold symmetry axis *c* (see Supplementary Fig. 1-a,b). The cell parameter reported in Supplementary Table 1 are taken from the original study in Ref.(27) of the main text. For the IXS experiment the crystal was oriented in the beam with respect to the conventional crystal cell, identifying the scattering plane (H 0 L). In that scattering plane the symmetry direction of the Brillouin zone (BZ) are written in the conventional cell basis (see Supplementary Tabs. 2 and 3).

**Cell Parameters**	
$a = 13.3796$ Å	$\alpha = 90°$
$b = 13.3796$ Å	$\beta = 90°$
$c = 9.7766$ Å	$\gamma = 90°$

Supplementary Table 1. Cell parameters. Parameter of the [VO(TPP)] tetragonal unit cell.

**Reciprocal lattice (HKL)**	**Reciprocal lattice (hkl)**
$R_1 = \left(\frac{2\pi}{a}, 0,0\right)$	$r_1 = \left(\frac{2\pi}{a}, \frac{2\pi}{a}, 0\right)$
$R_2 = \left(0, \frac{2\pi}{a}, 0\right)$	$r_2 = \left(\frac{2\pi}{a}, 0, \frac{2\pi}{c}\right)$
$R_3 = \left(0,0, \frac{2\pi}{c}\right)$	$r_3 = \left(0, \frac{2\pi}{a}, \frac{2\pi}{c}\right)$

Supplementary Table 2. Reciprocal lattice parameters. Definition of the reciprocal lattice vectors in the conventional (HKL) and primitive (hkl) cell basis.

**BZ Symmetry points**	r_i **basis**	R_i **basis**
$N = \left(0, \frac{1}{2}, 0\right)$	$\left(\frac{\pi}{a}, 0, \frac{\pi}{c}\right)$	$(H, 0, L)$
$K_x = \left(\frac{1}{4}, \frac{1}{4}, -\frac{1}{4}\right)$	$\left(\frac{\pi}{a}, 0,0\right)$	$(H, 0,0)$
$K_z = \left(\frac{-1}{4}, \frac{1}{4}, \frac{1}{4}\right)$	$\left(0,0, \frac{\pi}{c}\right)$	$(0,0, L)$

Supplementary Table 3. Brillouin zone symmetry points. Definition of the BZ symmetry points in the primitive and conventional cell basis, corresponding to the symmetry direction explored within the scattering plane.

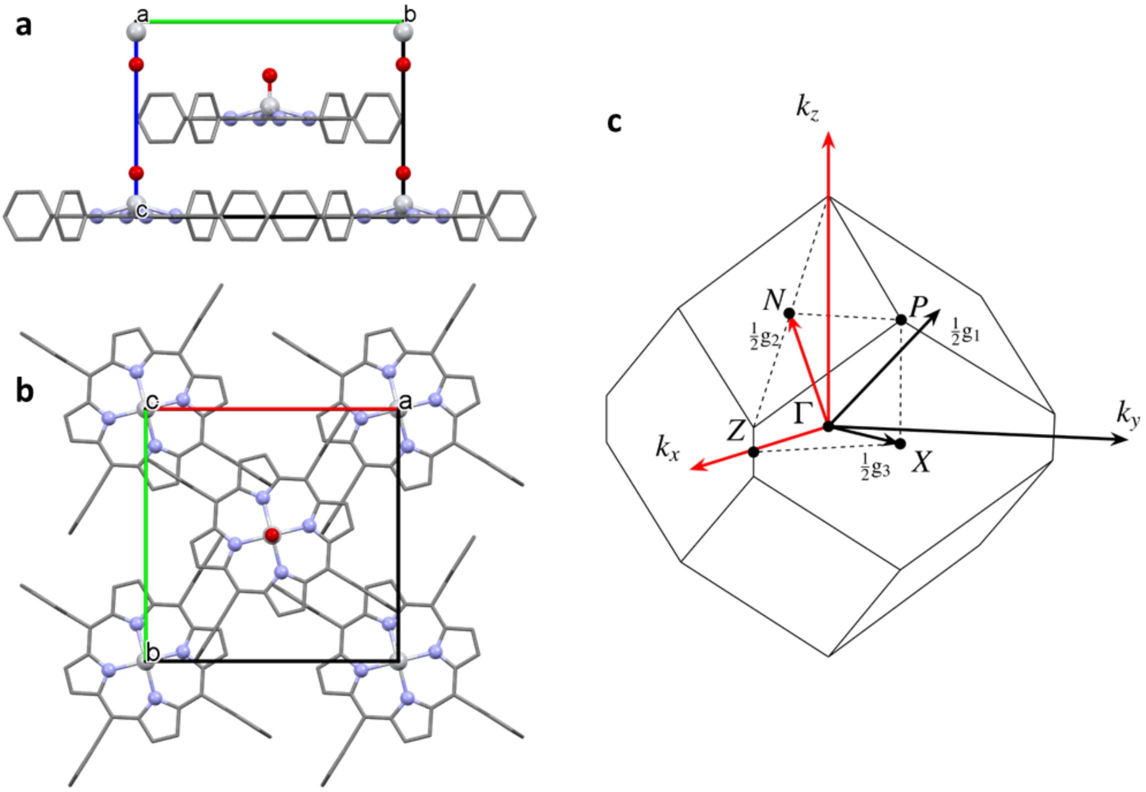

Supplementary Figure 1. Crystal packing and Brillouin Zone. a-**b** report views of the [VO(TPP)] crystal structure along the ***a*** and ***c*** axis respectively. In **c** we show the Brillouin zone, in which the symmetry direction probed in our experiment are highlighted (red arrows).

Supplementary Figures 2-7 - Phononic excitations intensities: ID28 vs simulations

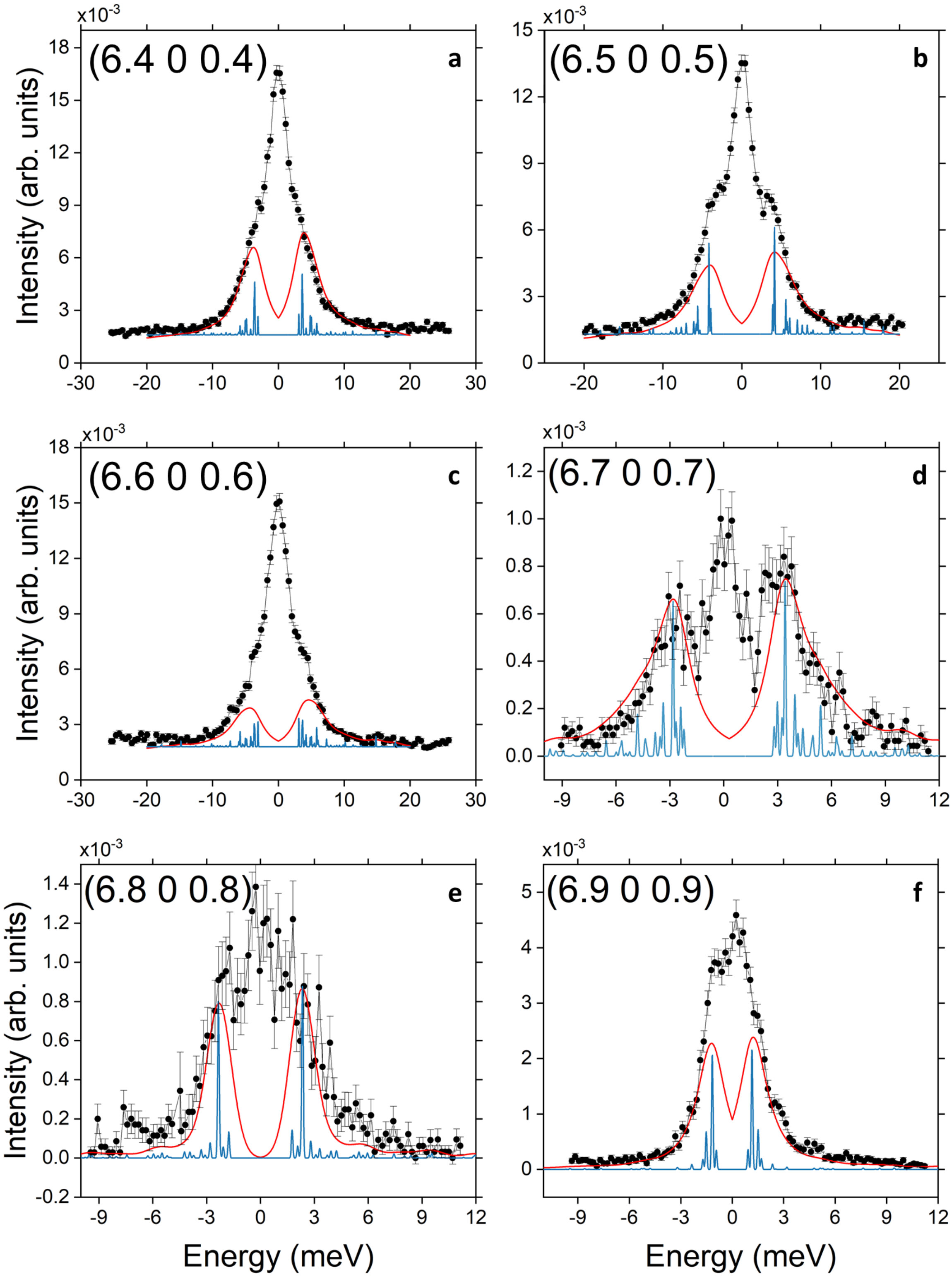

Supplementary Figure 2. ID28 vs simulations along $\Gamma - N$ from Bragg (6 0 0). Excitation intensities measured on ID28 (black scatters, with error bars representing the SE) along the $\Gamma - N$ symmetry direction at the specified $(6 + h0h)\ Q$ values. The experimental excitation intensities are compared with the calculated cross-section (red and light blue line) calculated with the experimental resolution (**a**-**c**: $\delta E = 3$ meV; **d**-**f**: $\delta E = 1.5$ meV) and with a 0.1 meV FWHM respectively. The elastic line is omitted for clarity.

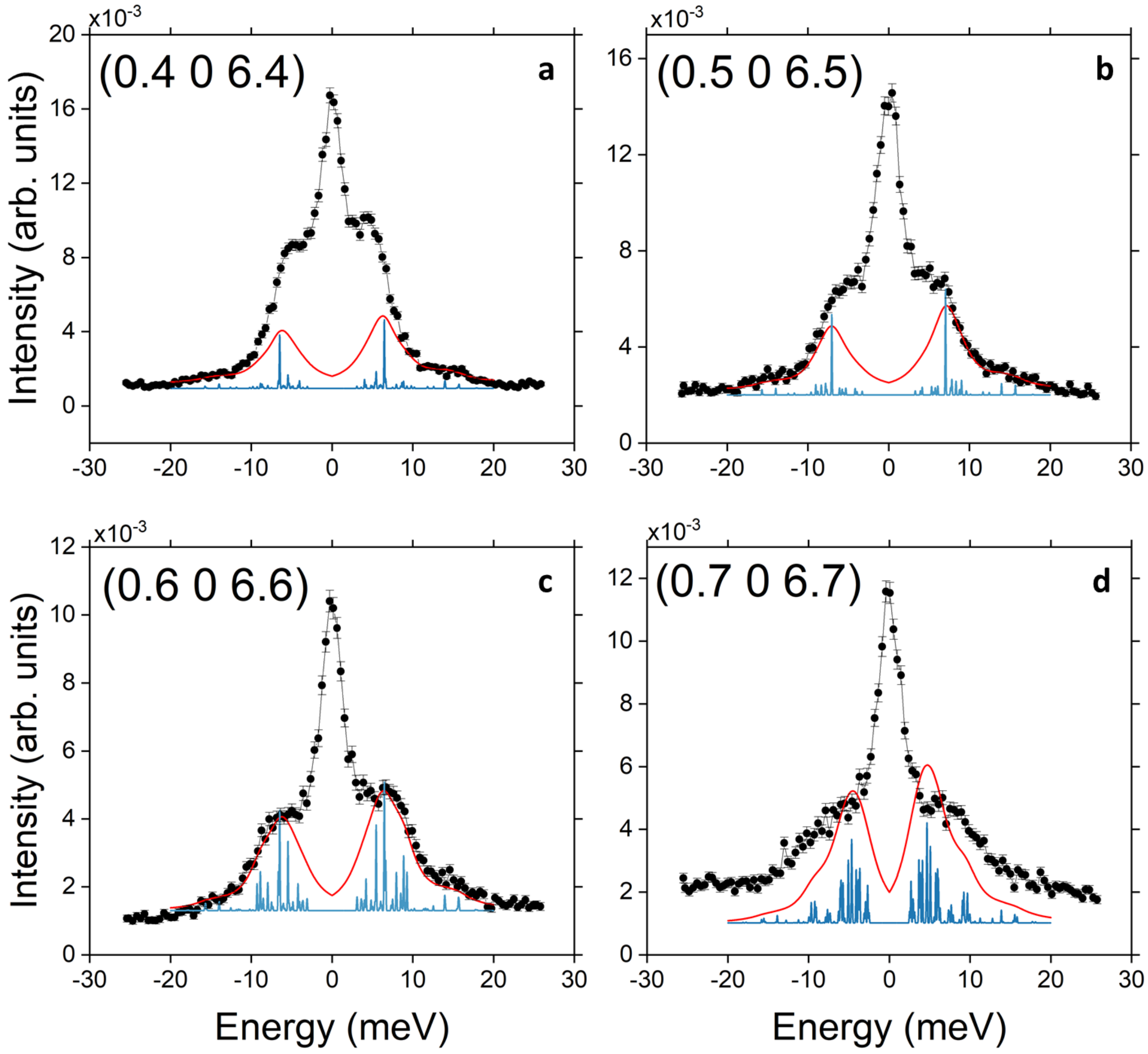

Supplementary Figure 3. ID28 vs simulations along $\Gamma - N$ from Bragg (0 0 6). Excitation intensities measured on ID28 (black scatters, with error bars representing the SE) along the $\Gamma - N$ symmetry direction at the specified $(h06+h)$ Q values. The experimental excitation intensities are compared with the calculated cross-section (red and light blue line) calculated with the experimental resolution (**a**-**d**: $\delta E = 3$ meV) and with a 0.1 meV FWHM respectively. The elastic line is omitted for clarity.

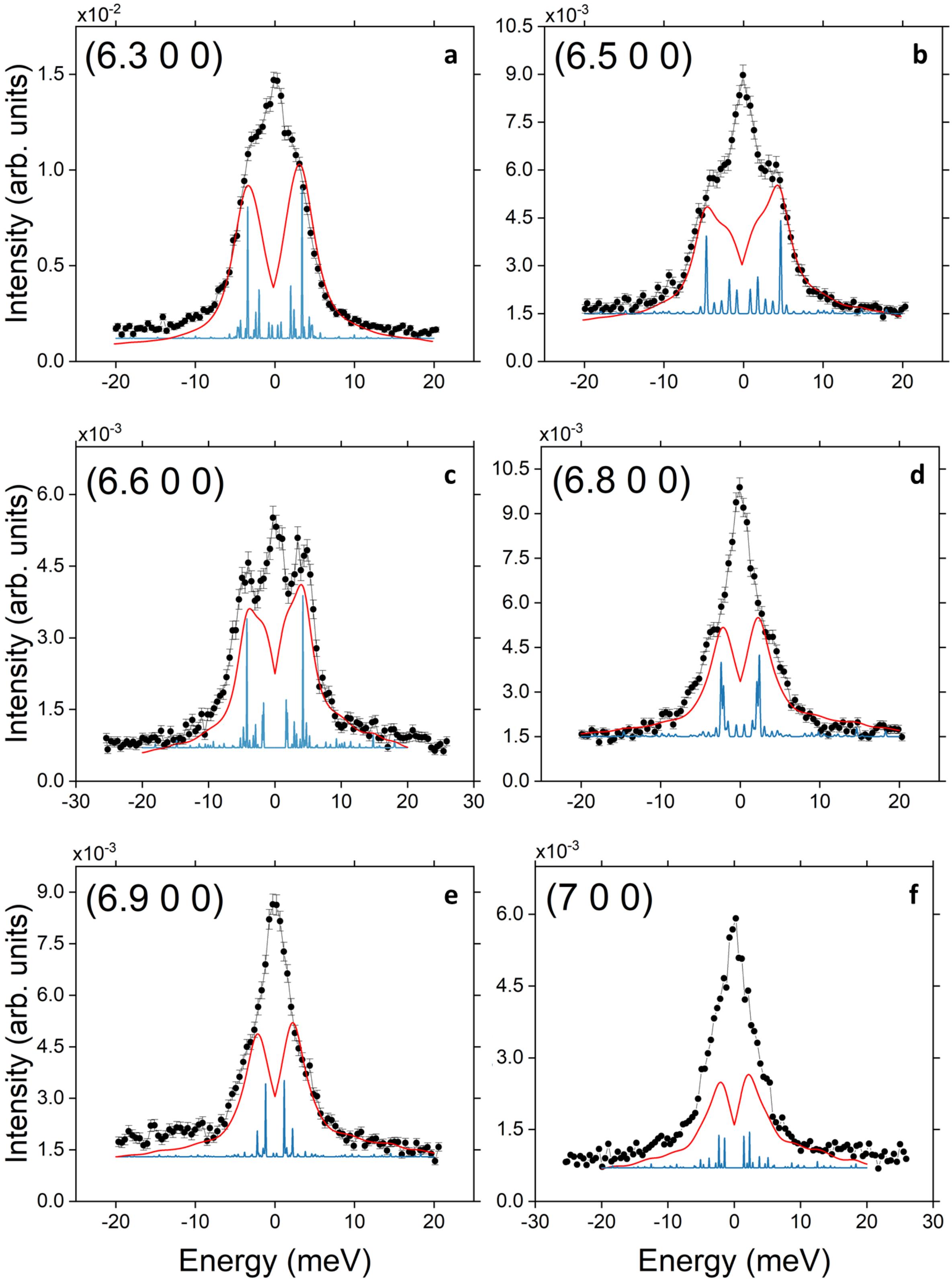

Supplementary Figure 4. ID28 vs simulations along $\Gamma - K_x$ longitudinal. Excitation intensities measured on ID28 (black scatters, with error bars representing the SE) for longitudinal scans along the $\Gamma - K_x$ symmetry direction at the specified $(6 + h\ 0\ 0)$ $\mathbf{Q}$ values. The experimental excitation intensities are compared with the calculated cross-section (red and light blue line) calculated with the experimental resolution (**a**-**f**: δE = 3 meV) and with a 0.1 meV FWHM respectively. The elastic line is omitted for clarity.

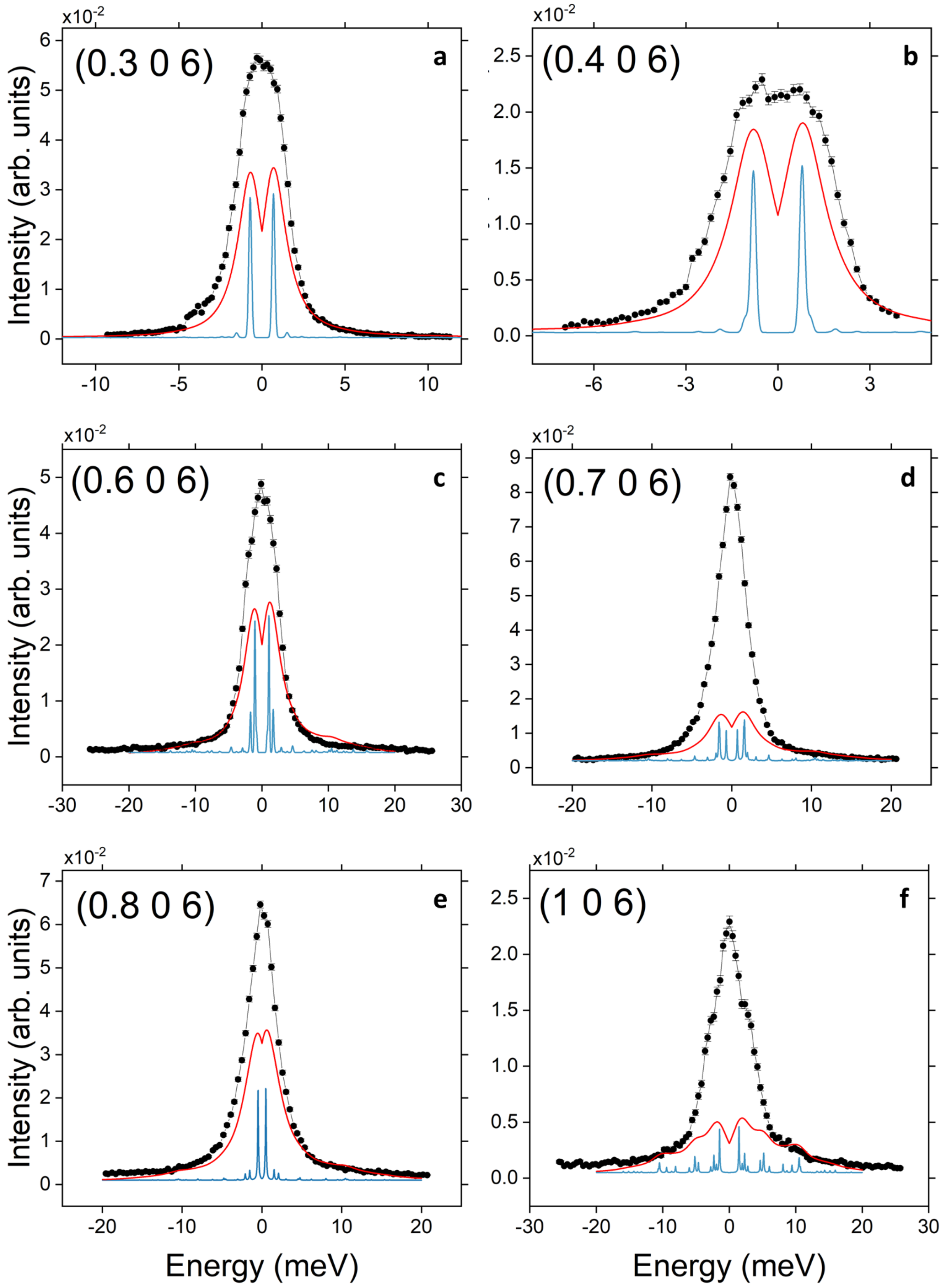

Supplementary Figure 5. ID28 vs simulations along $\Gamma - K_x$ transverse. Excitation intensities measured on ID28 (black scatters, with error bars representing the SE) for transverse scans along the $\Gamma - K_x$ symmetry direction at the specified $(\boldsymbol{h}\ \mathbf{0}\ \mathbf{6})$ $\mathbf{Q}$ values. The experimental excitation intensities are compared with the calculated cross-section (red and light blue line) calculated with the experimental resolution (**c-f**: δE = 3 meV; **a-b**: δE = 1.5 meV) and with a 0.1 meV FWHM respectively. The elastic line is omitted for clarity.

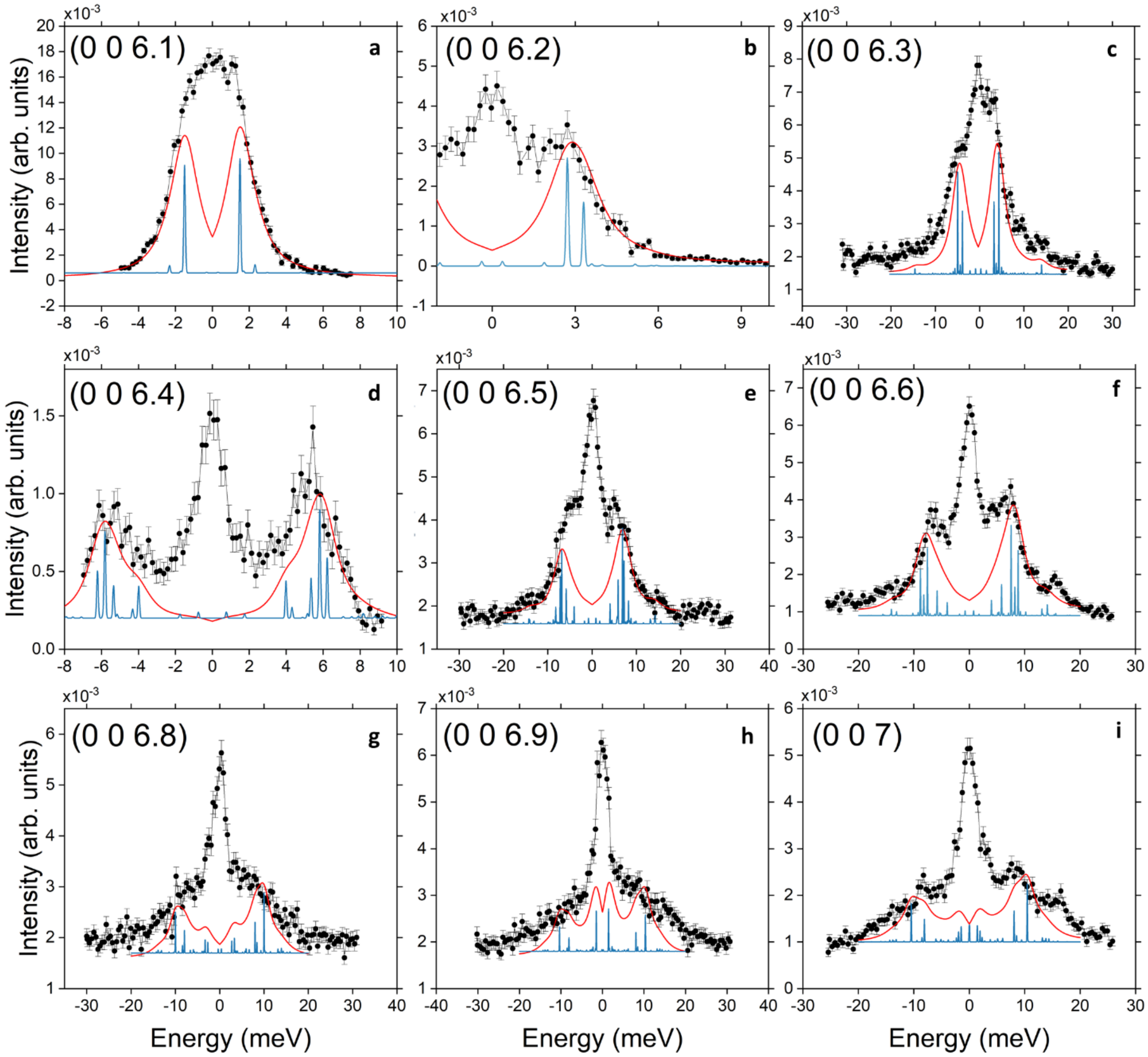

Supplementary Figure 6. ID28 vs simulations along $\Gamma - K_z$ longitudinal. Excitation intensities measured on ID28 (black scatters, with error bars representing the SE) for longitudinal scans along the $\Gamma - K_z$ symmetry direction at the specified $(0\ 0\ 6 + l)$ **Q** values. The experimental excitation intensities are compared with the calculated cross-section (red and light blue line) calculated with the experimental resolution (**c**,**e**-**i**: δE= 3 meV; **a**,**b**,**d**: δE = 1.5meV) and with a 0.1 meV FWHM respectively. The elastic line is omitted for clarity.

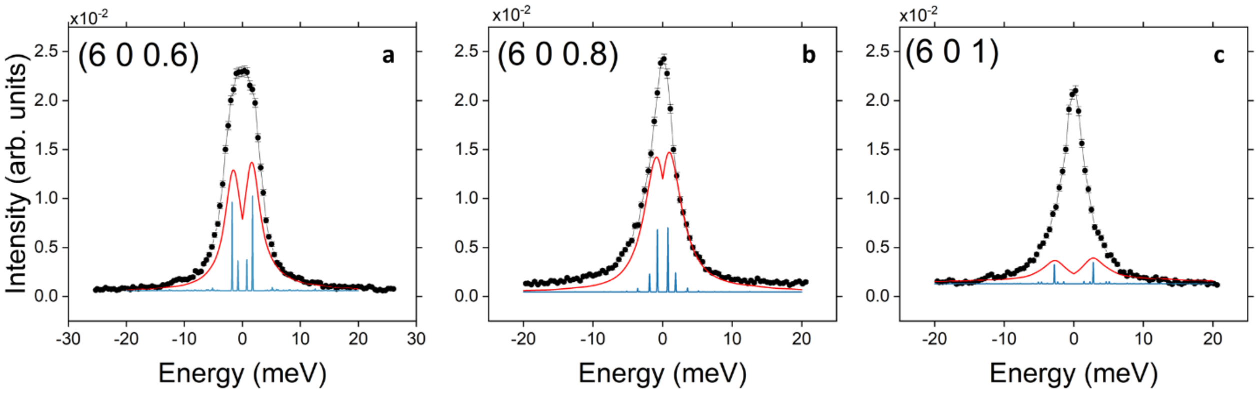

Supplementary Figure 7. ID28 vs simulations along $\Gamma - K_z$ transverse. Excitation intensities measured on ID28 (black scatters, with error bars representing the SE) for transverse scans along the $\boldsymbol{\Gamma - K_z}$ symmetry direction at the specified $(\mathbf{6\ 0}\ \boldsymbol{l})$ $\mathbf{Q}$ values. The experimental excitation intensities are compared with the calculated cross-section (red and light blue line) calculated with the experimental resolution (**a-c**: $\boldsymbol{\delta E}$ = 3 meV) and with a 0.1 meV FWHM respectively. The elastic line is omitted for clarity.

Supplementary Figure 8 - DFT calculations of phonon dispersions

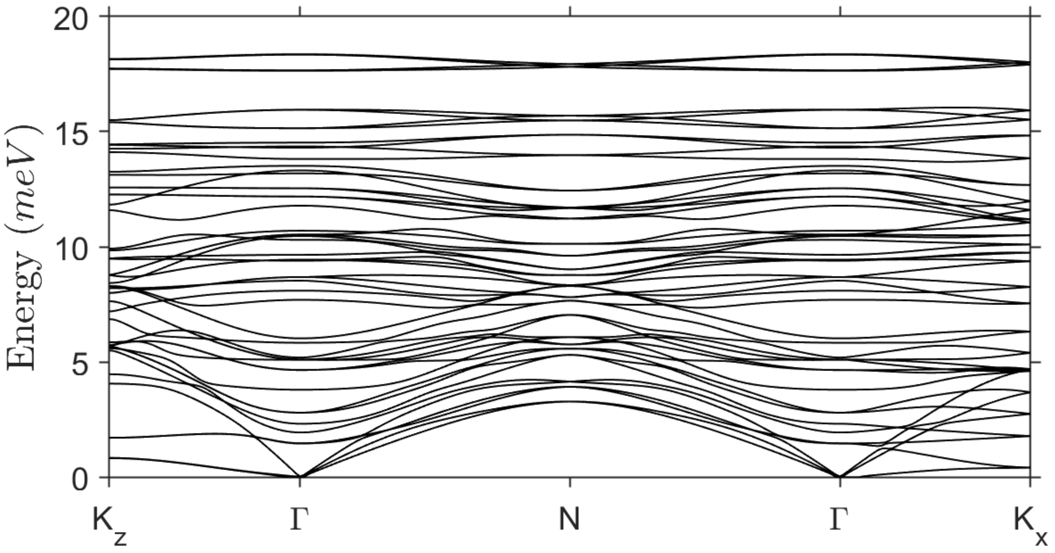

Supplementary Figure 8. DFT Phonon dispersions. Calculated low energy phonon dispersion (black lines) of [VO(TPP)] up to 20 meV. The K_z (0,0,0.5) $-\Gamma$(0,0,0) – N(0.5,0,0.5) – Γ(0,0,0) – K_x (0.5,0,0) path covers all the symmetry direction explored in the IXS experiment. The x-axis labelling highlight the symmetry points of the crystal Brillouin zone (see Supplementary Fig. 1-c).

Supplementary Note 2 – Computational workflow

- DFT optimization is carried out with CP2K by optimizing both the lattice cell and the atomic positions.
- Phonons are calculated by finite differentiating the forces with a two-point step of 0.01 Å
- A and g tensors are calculated for 2000 randomly distorted structures using the ORCA package. The computational detail is specified below.
- This is then used to train machine learning models in Python. The models with the best RMSE (Supplementary Figs. 9-11) are then used to calculate the first and second derivatives. The first and second derivatives for the first and second neighbours of Vanadium are computed both using machine learning and using DFT and the results are compared to ensure the quality of the coefficients calculated by machine learning (Supplementary Fig. 12).
- The obtained derivatives and phonon modes are then passed to MolForge to compute the decay of the magnetization along z or the xy plane (Supplementary Figs. 13-14). This is then fitted with exponential functions to obtain the relaxation times.

	**Cell Parameters**	
	Optimized structure	X-ray structure
a	13.2613 Å	13.3796 Å
b	13.2613 Å	13.3796 Å
c	9.6851 Å	9.7766 Å
α	90°	90°
β	90°	90°
γ	90°	90°
	**Bond length**	
	Optimized structure	X-ray structure
V=O	1.600 Å	1.630 Å
V-N	2.088 Å	2.104 Å

Supplementary Table 4. Optimized vs X-ray geometry comparison. Comparison between the cell parameters and bond lengths of the periodic DFT optimized [VO(TPP)] unit cell and the X-ray unit cell.

All the neural networks used in this work are made with the Tensorflow library and Keras API. The simple model has an input layer with 234 nodes, which translates to 3N Cartesian coordinates with N being the number of atoms in the molecule, and an output layer with 9 nodes, corresponding to the Cartesian tensor components of **A** or **g**. We tested the model with different numbers of hidden layers and the number of nodes in each hidden layer to obtain the optimized model for learning **A** and **g** tensors.

Each model is trained on a training set of 1600 configurations of randomly distorted [VO(TPP)] molecules. The random distortions are within the range of ±0.05 Å, applied to the equilibrium structure of [VO(TPP)]. The regularization hyperparameters are optimized for each model using a validation set of 158 configurations and then, each model's performance is evaluated with a test set of 200 configurations. DFT calculations are performed to obtain the needed data for the data set. A DKH-def2-TZVPP basis is used for V, N, and O atoms, and a DKH-def2-SVP basis is used for C and H atoms. All basis sets are decontracted and RIJCOSX is used as approximation for Coulomb and HF exchange. All DFT calculations are done using the hybrid functional PBE0 and a tight convergence in

the ORCA software. Supplementary Figure 9 shows the learning curve of different models for the **A** and **g** tensors.

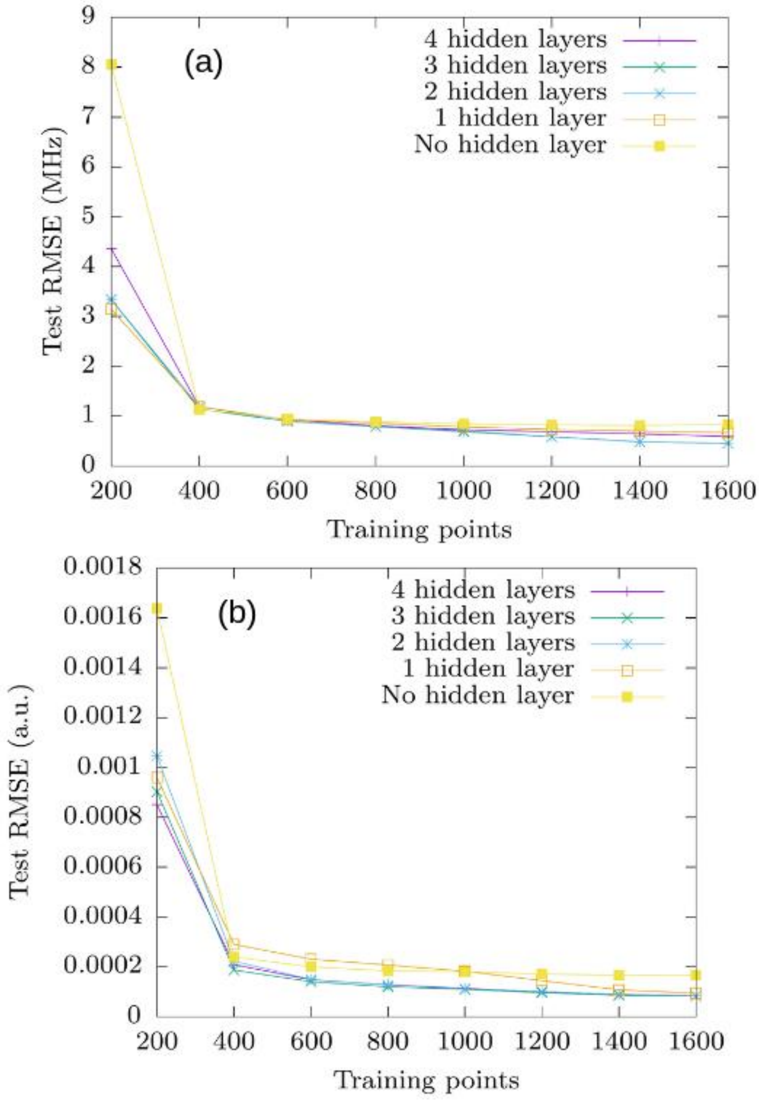

Supplementary Figure 9. Learning curves for the A and g tensors. The learning curve of machine learning models with different numbers of hidden layers for learning **A** tensor (**a**) and **g** tensor (**b**). The purple line and plus symbols report the learning curve of the model with 4 hidden layers; the green line and cross symbols report the learning curve of the model with 3 hidden layers; the blue line and asterisks report the learning curve of the model with 2 hidden layers; the orange line and unfilled squares report the learning curve of the model with 1 hidden layer; the yellow line and filled squares report the learning curve of the model with no hidden layer.

The models that performed the best for **A** and **g** tensors are chosen, with an error of 0.453 MHz for the **A** tensor and 8.236×10^{-5} for the **g** tensor (Supplementary Figs. 10-11).

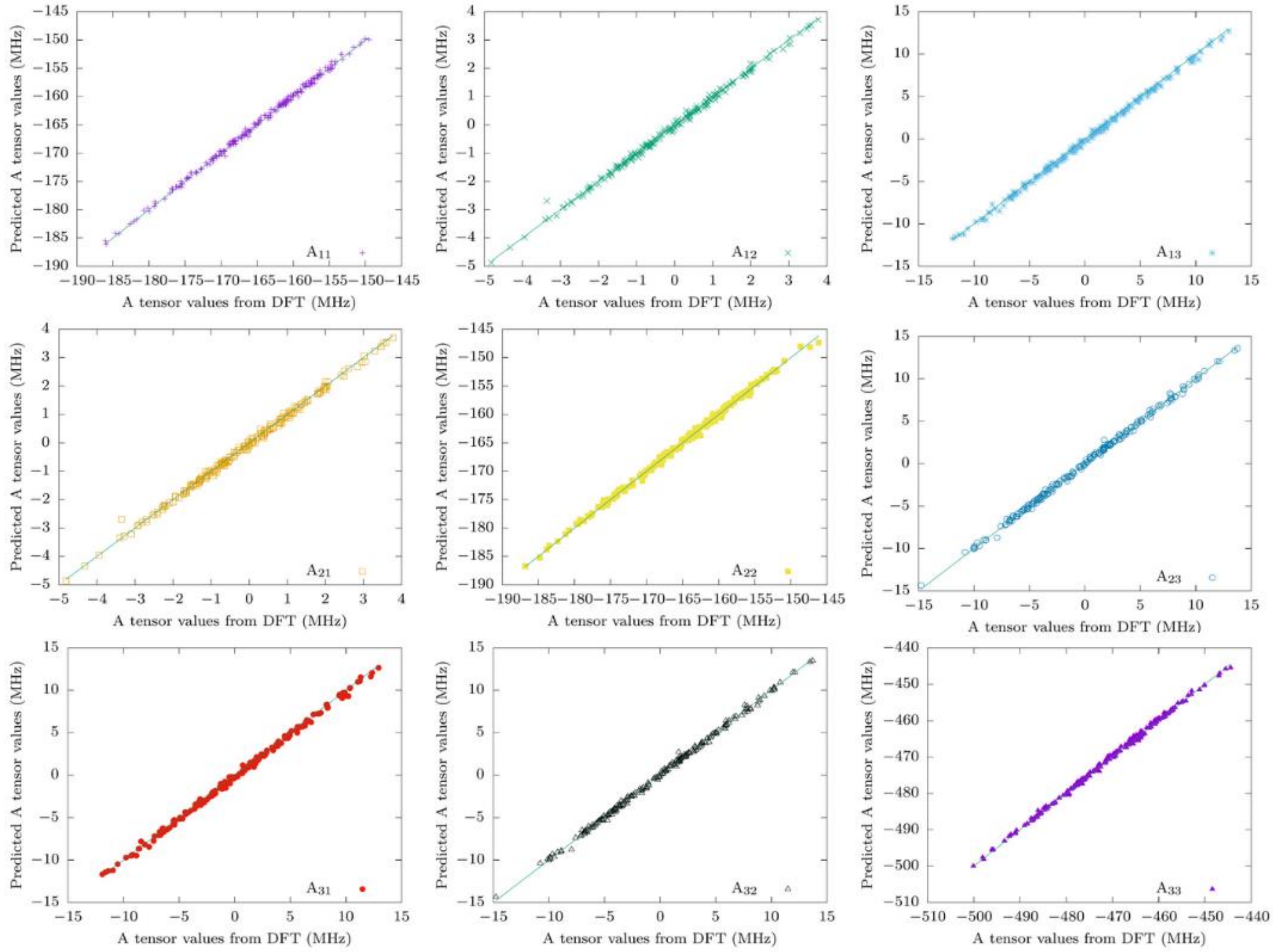

Supplementary Figure 10. Machine learning vs DFT values for the A tensor. Plot of the predicted value with machine learning against the calculated value from DFT for each Cartesian **A** tensor component using the model with the lowest error.

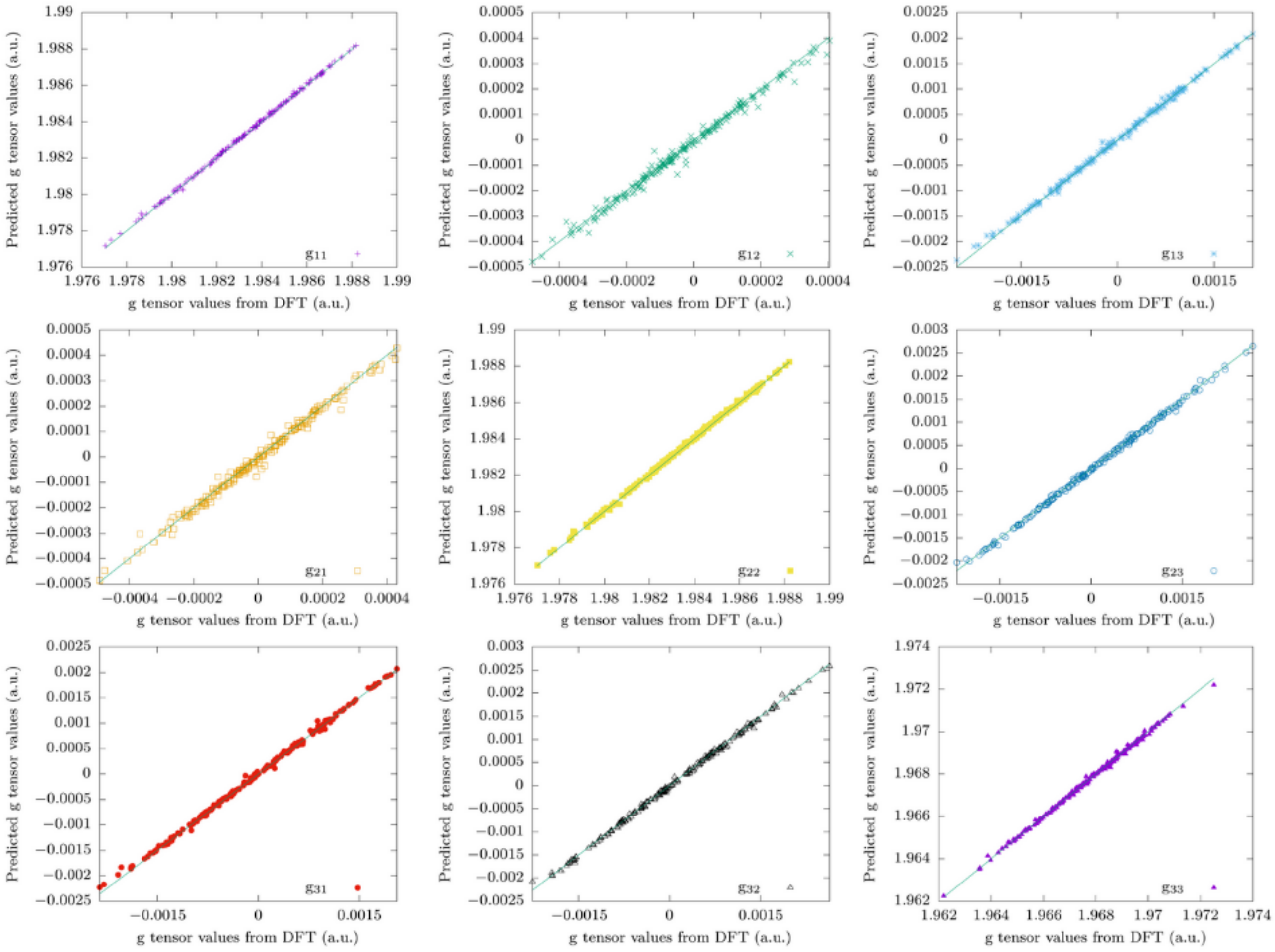

Supplementary Figure 11. Machine learning vs DFT values for the g tensor. Plot of the predicted value with machine learning against the calculated value from DFT for each Cartesian **g** tensor component using the model with the lowest error.

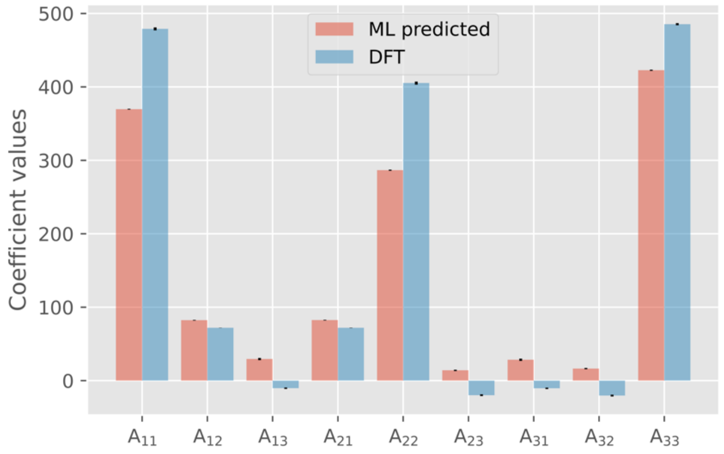

Supplementary Figure 12. Machine learning vs DFT computed second order derivatives. Predicted second order derivatives computed with machine learning against the calculated values from DFT for the **A** tensor values when displacing atom 38 (N) along y and atom 75 (N) along x.

Supplementary Note 3 – Spin relaxation simulations

The first- and second-order derivatives, $V_{\alpha\mathbf{q}}$ and $V_{\alpha\mathbf{q}\beta\mathbf{q}'}$, are calculated numerically from the **A** and **g** tensors computed with machine learning. A grid of 6×6 points is used to calculate each derivative and it is fitted using a 2D second-order polynomial expression with the linear least square method. The derivatives are then used to simulate the spin relaxation process of [VO(TPP)] and calculate the spin-phonon relaxation time T_1 and T_2. We consider the quadratic term of the spin-phonon coupling Hamiltonian, which corresponds to a two-phonon process. The spin density $\rho^s_{ab}(t)$ of the system evolves in time as

$$\frac{d\rho^s_{ab}(t)}{dt} = \sum_{cd} e^{i(\omega_{ac}+\omega_{db})t} R^{\text{2-ph}}_{ab,cd}\rho^s_{cd}(t) \qquad \text{(S1)}$$

in which $\omega_{ab} = (E_a - E_b)/\hbar$ and the Redfield operator $R_{ab,cd}$ is:

$$\begin{aligned} R^{\text{2-ph}}_{ab,cd} = -\frac{\pi}{4\hbar^2} \sum_{\alpha\mathbf{q}\geq\beta\mathbf{q}'} \Bigg\{ & \sum_j \delta_{bd} V^{\alpha\mathbf{q}\beta\mathbf{q}'}_{aj} V^{\alpha-\mathbf{q}\beta-\mathbf{q}'}_{jc} G^{\text{2-ph}}(\omega_{jc}, \omega_{\alpha\mathbf{q}}, \omega_{\beta\mathbf{q}'}) \\ & - V^{\alpha\mathbf{q}\beta\mathbf{q}'}_{ac} V^{\alpha-\mathbf{q}\beta-\mathbf{q}'}_{db} G^{\text{2-ph}}(\omega_{ac}, \omega_{\alpha\mathbf{q}}, \omega_{\beta\mathbf{q}'}) \\ & - V^{\alpha\mathbf{q}\beta\mathbf{q}'}_{ac} V^{\alpha-\mathbf{q}\beta-\mathbf{q}'}_{db} G^{\text{2-ph}}(\omega_{bd}, \omega_{\alpha\mathbf{q}}, \omega_{\beta\mathbf{q}'}) \\ & + \sum_j \delta_{ca} V^{\alpha\mathbf{q}\beta\mathbf{q}'}_{dj} V^{\alpha-\mathbf{q}\beta-\mathbf{q}'}_{jb} G^{\text{2-ph}}(\omega_{jd}, \omega_{\alpha\mathbf{q}}, \omega_{\beta\mathbf{q}'}) \Bigg\}, \end{aligned} \qquad \text{(S2)}$$

where

$$\begin{aligned} G^{\text{2-ph}}(\omega_{ij}, \omega_{\alpha\mathbf{q}}, \omega_{\beta\mathbf{q}'}) = {} & \delta(\omega_{ij} - \omega_{\alpha\mathbf{q}} - \omega_{\beta\mathbf{q}'})\, \bar{n}_{\alpha\mathbf{q}}\bar{n}_{\beta\mathbf{q}'} \\ & + \delta(\omega_{ij} + \omega_{\alpha\mathbf{q}} + \omega_{\beta\mathbf{q}'})(\bar{n}_{\alpha\mathbf{q}} + 1)(\bar{n}_{\beta\mathbf{q}'} + 1) \\ & + \delta(\omega_{ij} + \omega_{\alpha\mathbf{q}} - \omega_{\beta\mathbf{q}'})(\bar{n}_{\alpha\mathbf{q}} + 1)\bar{n}_{\beta\mathbf{q}'} \\ & + \delta(\omega_{ij} - \omega_{\alpha\mathbf{q}} + \omega_{\beta\mathbf{q}'})\bar{n}_{\alpha\mathbf{q}}(\bar{n}_{\beta\mathbf{q}'} + 1). \end{aligned} \qquad \text{(S3)}$$

The Dirac delta function $\delta(x)$ is approximated using a Gaussian:

$$\delta(x) \sim \frac{1}{\sigma\sqrt{\pi}} e^{-x^2/\sigma^2}$$

The Redfield operator is converged to the harmonic limit by numerically taking the double limit

$$R_{ab,cd} \propto \int_{BZ} \delta(\omega - \omega_q) dq = \lim_{\sigma\to 0} \lim_{q\to\infty} \sum_q \frac{1}{\sigma\sqrt{\pi}} e^{-(\omega-\omega_q)^2/\sigma^2} \qquad \text{(S4)}$$

The smearing σ in Eq. S4 and the q-point grid are varied to obtain the most accurate T_1 and T_2 profiles with respect to temperature.

T_1 and T_2 are calculated by fitting the decay of the magnetization (Supplementary Figs. 13) with a double exponential if it was computed with the **A** tensor or with a single exponential if it was computed with the **g** tensor. Supplementary Figures 14-15 show the relaxation time T_1 calculated

from **A** tensor and Supplementary Figs. 16-17 show the relaxation time T_1 calculated from **g** tensor. Supplementary Figs. 18 and 19 show the T_2 calculated from **A** and **g** tensor, respectively.

**Temp. (K)**	**Expt. T_1 (μs)**	**Temp. (K)**	**Sim. T_1 (μs)**	**No low vib. T_1 (μs)**
5	1411	15	143.3	4265300.0
10	1343	17	112.9	2252090.0
16	943	20	75.8	1060150.0
25	456	25	50.9	413155.0
35	344	30	36.4	209089.0
40	227	40	21.0	84527.8
50	95	50	13.6	46334.7
60	51.1	75	5.7	16720.1
70	36.1	100	3.3	8802.2
80	21.8	200	0.8	2048.3
100	11.3	300	0.4	896.5
140	2.25			
200	1.024			
288	0.408			

Supplementary Table 5. Spin-phonon relaxation time. The computed spin-phonon relaxation time with respect to temperature in Fig. 5. The experimental values are obtained from Ref. (27) of the main text.

**Temp. (K)**	**Expt. T_2 (μs)**	**Temp. (K)**	**Sim. T_2 (μs)**
5	1.08	15	155.5
10	1.147	17	119.9
16	1.116	20	91.8
25	1.146	25	58.3
50	0.976	30	39.2
60	1.063	40	19.9
70	0.852	50	11.4
100	0.644	75	6.1
140	0.382	100	2.9
200	0.213	200	0.9
250	0.149	300	0.4
288	0.143		

Supplementary Table 6. Decoherence time. The computed decoherence time with respect to temperature in Fig. 5. The experimental values are obtained from Ref. (27) of the main text.

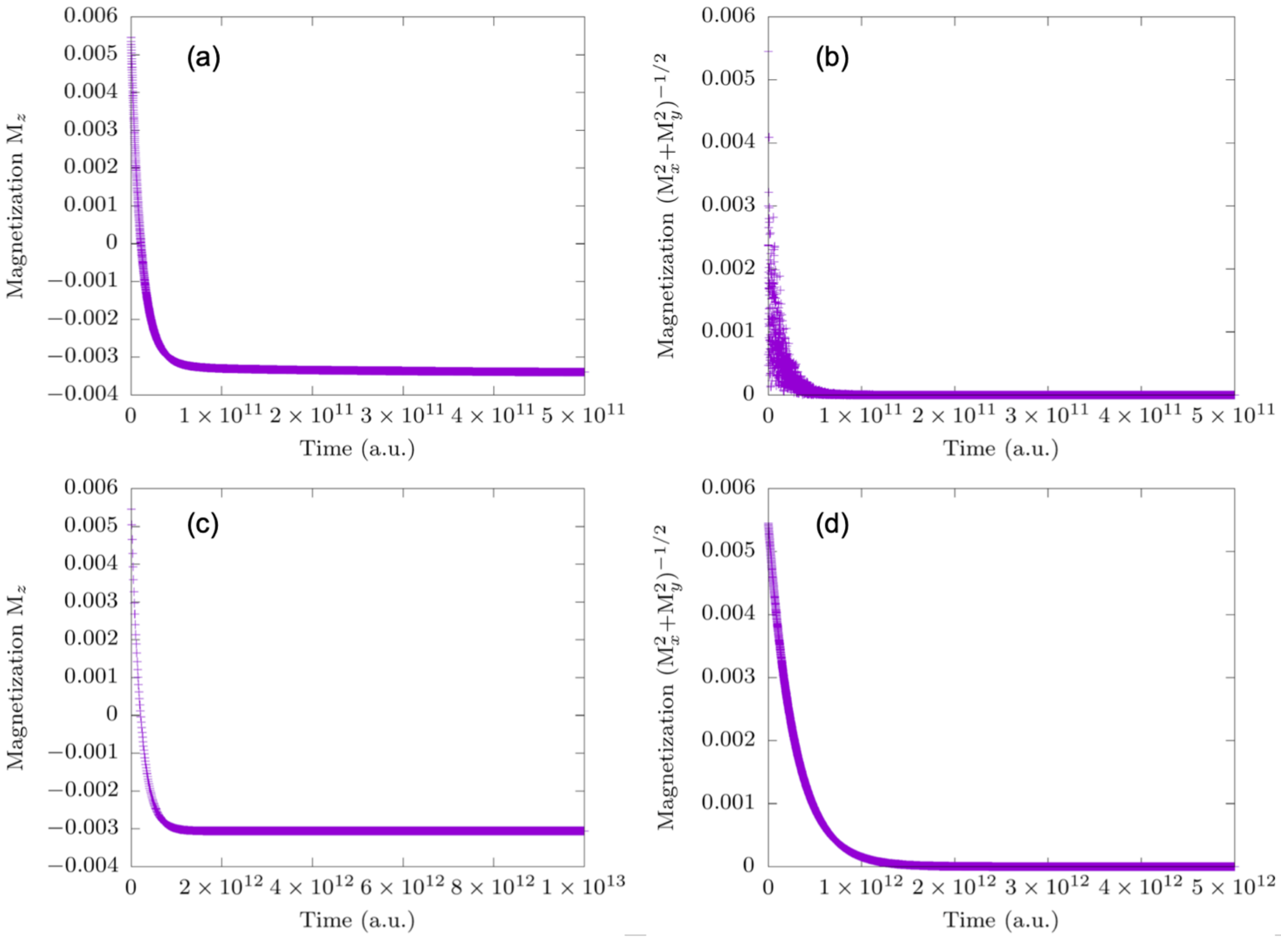

Supplementary Figure 13. Decay of the magnetization. Plot of the decay of $M_z(t)$ calculated from **A** tensor (**a**), $\left(M_x^2 + M_y^2\right)^{1/2}$ calculated from **A** tensor (**b**), $M_z(t)$ calculated from **g** tensor (**c**), $\left(M_x^2 + M_y^2\right)^{1/2}$ calculated from **g** tensor (**d**) for [VO(TPP)] at 20K, calculated with smear 10 and a 1×1×1 q-point grid.

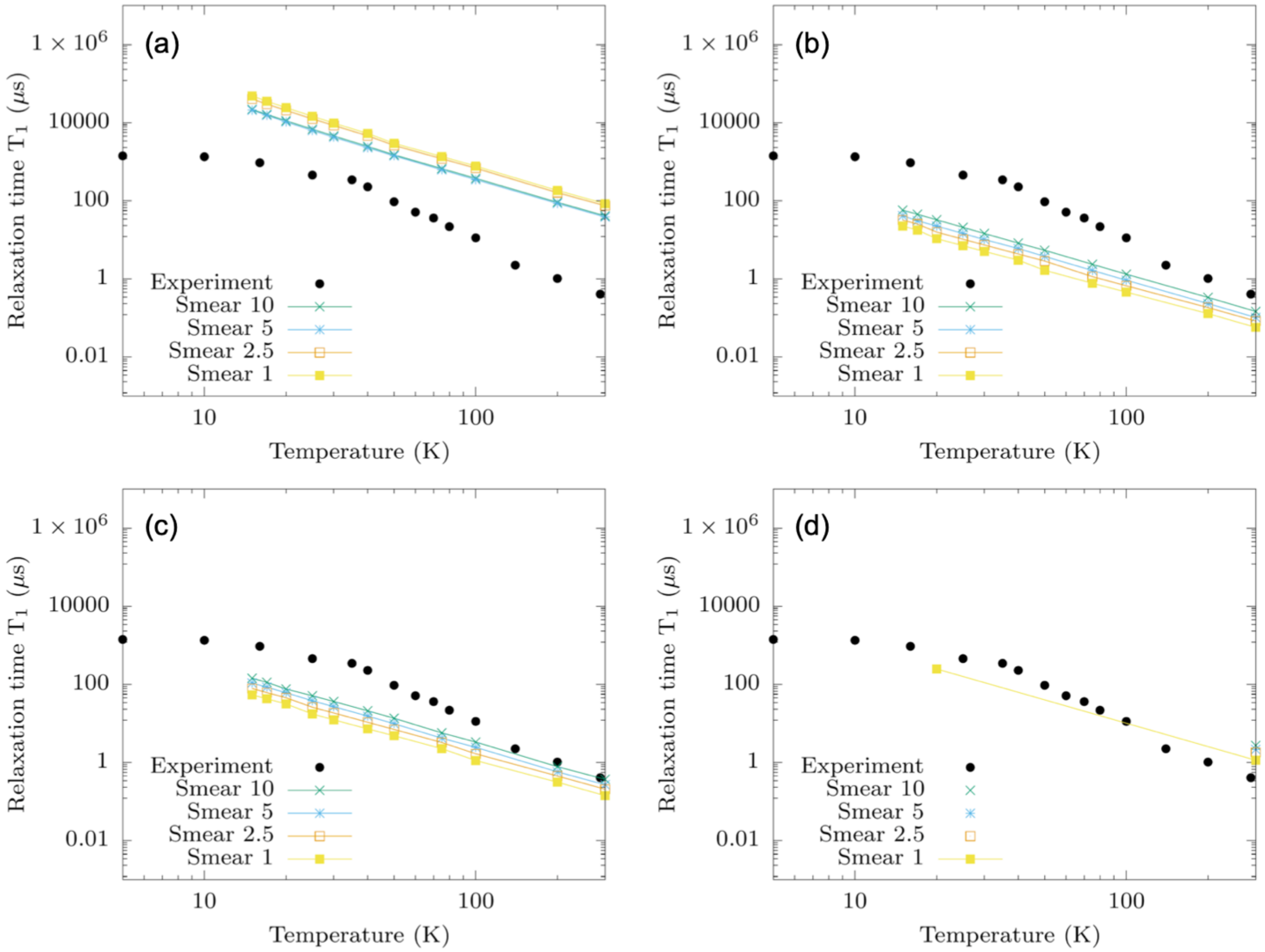

Supplementary Figure 14. Relaxation time T_1 calculated from A tensor. The T_1 against temperature profile of [VO(TPP)] calculated using **A** tensor with different smearing and 1×1×1 (**a**), 2×2×2 (**b**), 4×4×4 (**c**), and 6×6×6 (**d**) q-point grid. The green line and cross symbols report T_1 values calculated with smear 10; the blue line and asterisks report T_1 values calculated with smear 5; the orange line and unfilled squares report T_1 values calculated with smear 2.5; and the yellow line and filled squares report T_1 values calculated with smear 1. Black circles report the inversion recovery experimental results of Ref. (27) of the main text (error bars are within the size of the symbols).

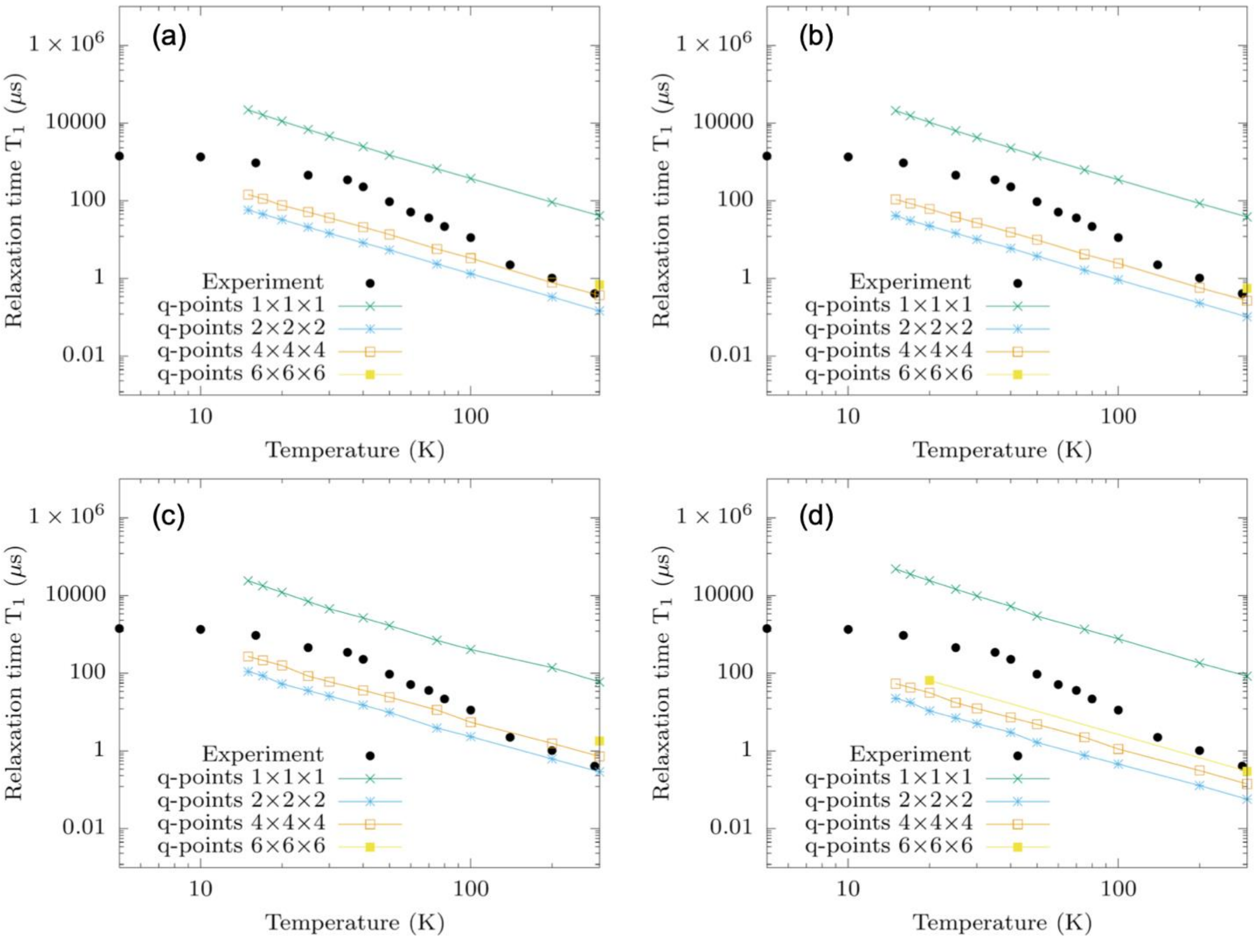

Supplementary Figure 15. Relaxation time T_1 calculated from A tensor with different k-points grid. The T_1 against temperature profile of [VO(TPP)] calculated using **A** tensor with different k-point grids and smear 10 (**a**), smear 5 (**b**), smear 2.5 (**c**), and smear 1 (**d**). The green line and cross symbols report T_1 values calculated with a q-point grid of 1×1×1; the blue line and asterisks report T_1 values calculated with a q-point grid of 2×2×2; the orange line and unfilled squares report T_1 values calculated with a q-point grid of 4×4×4; the yellow line and filled squares report T_1 values calculated with a q-point grid of 6×6×6. In d, the unfilled circle represents the T_1 values calculated with a q-point grid of 8×8×8. Black circles report the inversion recovery experimental results of Ref.(27) of the main text (error bars are within the size of the symbols).

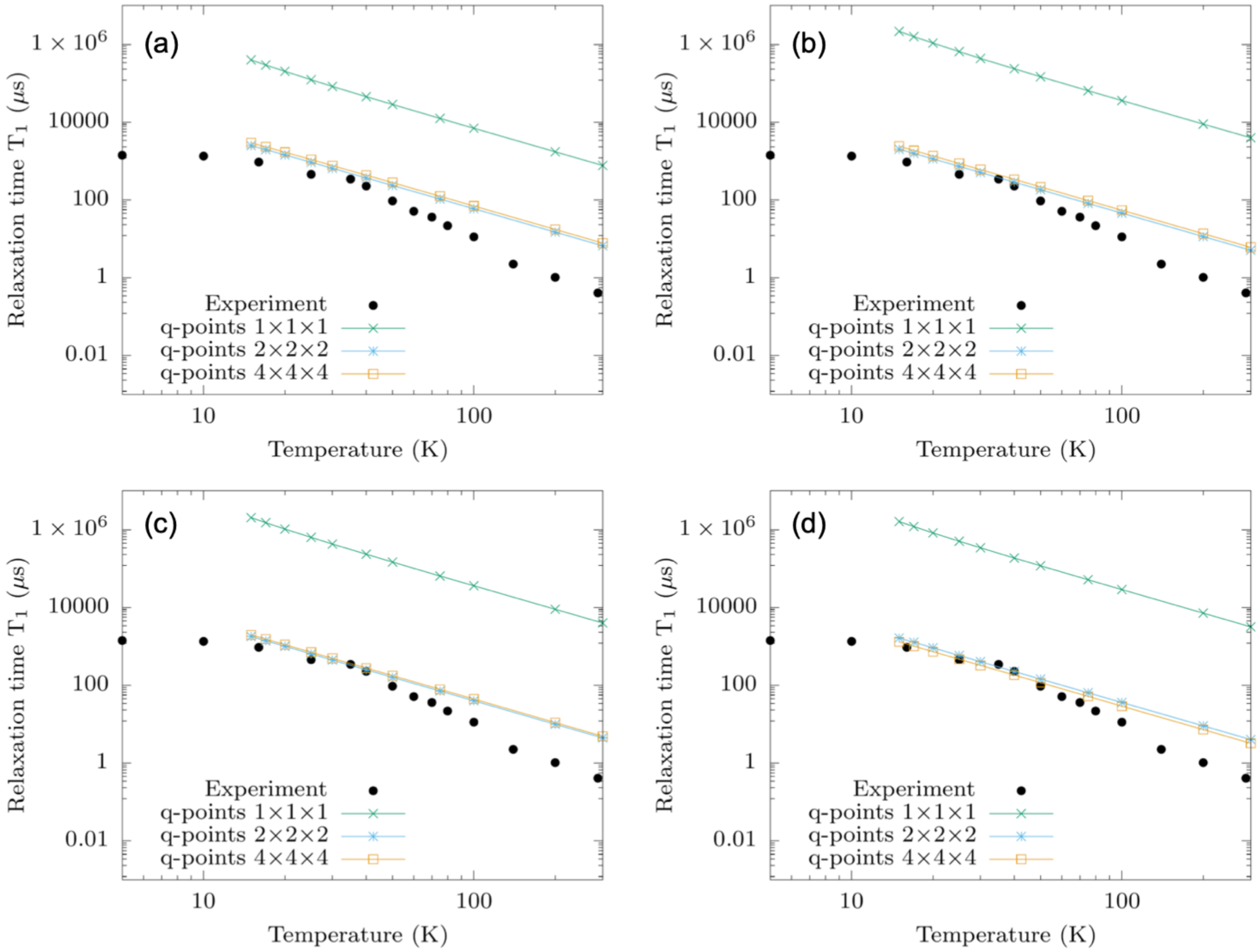

Supplementary Figure 16. Relaxation time T_1 calculated from g tensor. The T_1 against temperature profile of [VO(TPP)] calculated using **g** tensor with different q-point grids and smear 10 (**a**), smear 5 (**b**), smear 2.5 (**c**), and smear 1 (**d**). The green line and cross symbols report T_1 values calculated with a q-point grid of 1×1×1; the blue line and asterisks report T_1 values calculated with a q-point grid of 2×2×2; the orange line and unfilled squares report T_1 values calculated with a q-point grid of 4×4×4. Black circles report the inversion recovery experimental results of Ref.(27) of the main text (error bars are within the size of the symbols).

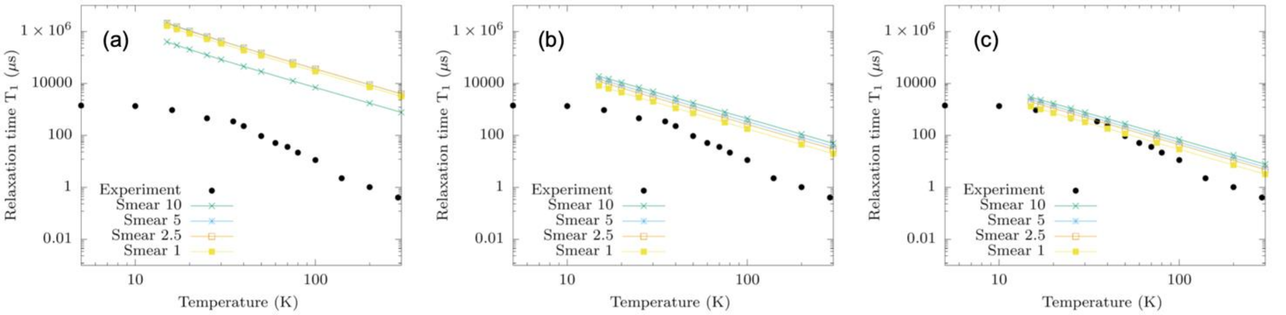

Supplementary Figure 17. Relaxation time T_1 calculated from g tensor with different k-points grid. The T_1 against temperature profile of [VO(TPP)] calculated using **g** tensor with different smearing and 1×1×1 (**a**), 2×2×2 (**b**) and 4×4×4 (**c**) q-point grid. The green line and cross symbols report T_1 values calculated with smear 10; the blue line and asterisks report T_1 values calculated with smear 5; the orange line and unfilled squares report T_1 values calculated with smear 2.5; the yellow line and filled squares report T_1 values calculated with smear 1. Black circles report the inversion recovery experimental results of Ref.(27) of the main text (error bars are within the size of the symbols).

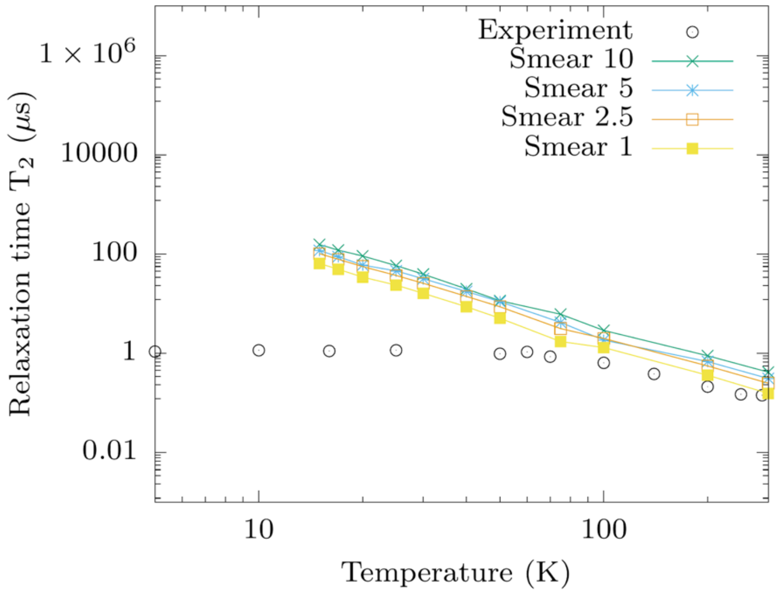

Supplementary Figure 18. Relaxation time T_2 calculated from A tensor. The T_2 against temperature profile of [VO(TPP)] calculated using **A** tensor with different smearing. The green line and cross symbols report T_2 values calculated with smear 10; the blue line and asterisks report T_2 values calculated with smear 5; the orange line and unfilled squares report T_2 values calculated with smear 2.5; the yellow line and filled squares report T_2 values calculated with smear 1. Empty circles report the Hahn echo experimental results of Ref.(27) of the main text (error bars are within the size of the symbols).

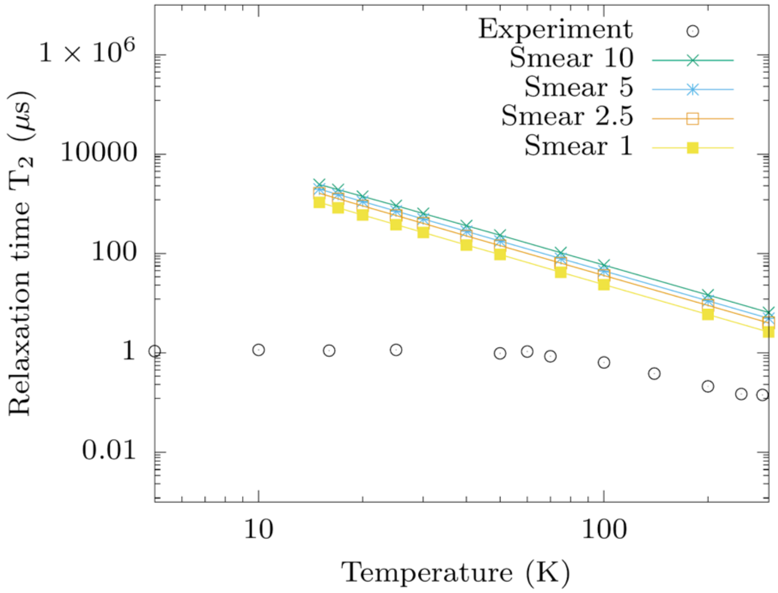

Supplementary Figure 19. Relaxation time T_2 calculated from g tensor. The T_2 against temperature profile of [VO(TPP)] calculated using **g** tensor with different smearing. The green line and cross symbols report T_2 values calculated with smear 10; the blue line and asterisks report T_2 values calculated with smear 5; the orange line and unfilled squares report T_2 values calculated with smear 2.5; the yellow line and filled squares report T_2 values calculated with smear 1. Empty circles report the Hahn echo experimental results of Ref. (27) of the main text (error bars are within the size of the symbols).

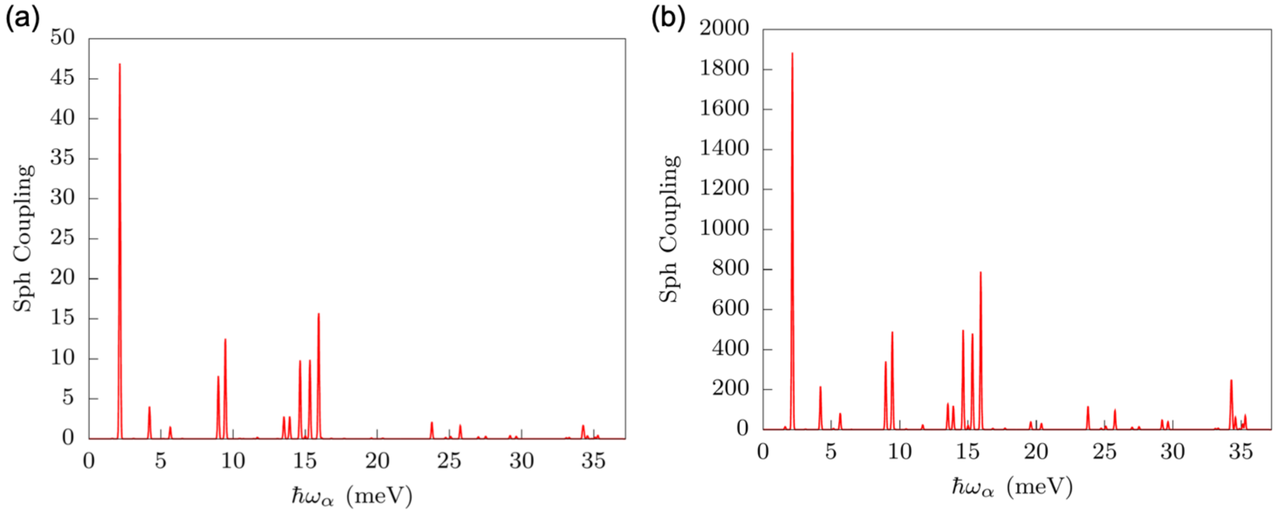

Supplementary Figure 20. Spin-phonon coupling distribution of phonon modes with low frequencies. The spin-phonon coupling distribution computed from the first order derivative of **A** tensor (**a**) and **g** tensor (**b**) are shown in read for phonon modes with low frequencies at the Γ point.

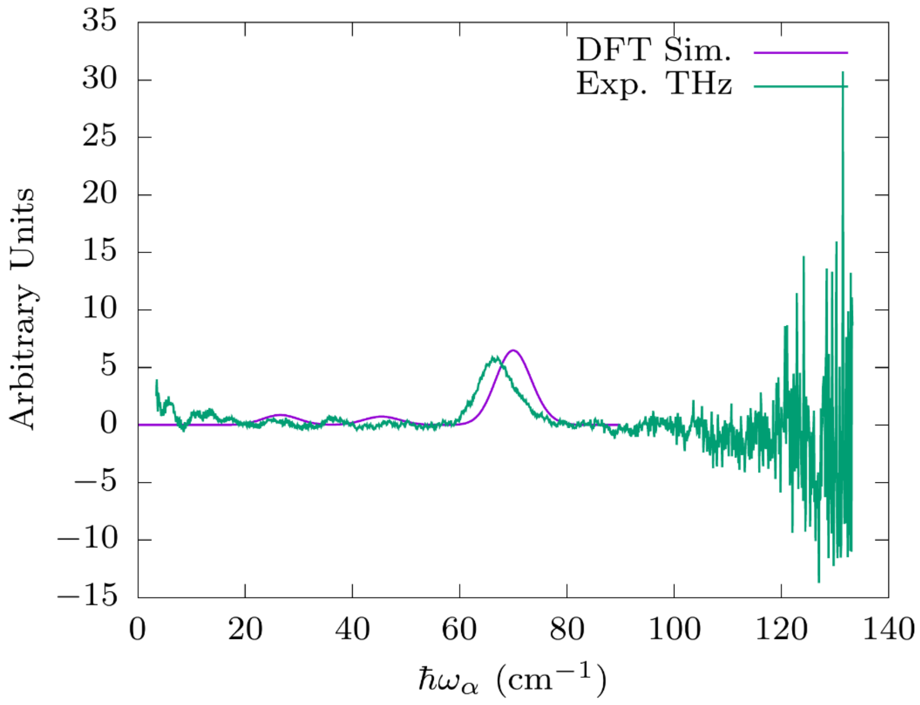

Supplementary Figure 21. IR spectrum. Experimental IR spectrum of [VO(TPP)] (green line, see Ref.(27) of the main text) compared with Γ-point simulation (purple line), reproducing the feature around 70 cm^{-1}.

Supplementary Note 4 – Spin-phonon pure dephasing time

Redfield relaxation theory predicts

$$\frac{1}{T_2} = \frac{1}{2T_1} + \frac{1}{T_2^*},$$

where T_2^* is the aforementioned pure dephasing contribution. This relation is true for both linear and quadratic coupling, but in the former case the contribution vanishes.

The explicit expression for the dephasing rate reads

$$\frac{1}{T_2^*} \propto -2V_{aa}V_{bb}G^{1-ph}(0,\omega_\alpha) + V_{aa}V_{aa}G^{1-ph}(0,\omega_\alpha) + V_{bb}V_{bb}G^{1-ph}(0,\omega_\alpha)$$

and

$$\frac{1}{T_2^*} \propto -2V_{aa}V_{bb}G^{2-ph}(0,\omega_\alpha,\omega_\beta) + V_{aa}V_{aa}G^{2-ph}(0,\omega_\alpha,\omega_\beta) + V_{bb}V_{bb}G^{2-ph}(0,\omega_\alpha,\omega_\beta),$$

for one- and two-phonon relaxation at the density matrix second-order time-dependent perturbation theory level, respectively.

The function $G^{1-ph}(\omega,\omega_\alpha) = n_\alpha\delta(\omega-\omega_\alpha) + (n_\alpha+1)\delta(\omega+\omega_\alpha)$ vanishes for $\omega = 0$ as there are no phonons available at zero frequency. The only exception is represented by the three acoustic branches, which however correspond to rigid translations of the entire crystal and do not couple with spin. As a consequence, the one-phonon contribution to pure dephasing is zero.

However, the function $G^{2-ph}(\omega,\omega_\alpha,\omega_\beta) = n_\alpha(n_\beta+1)\delta(\omega-\omega_\alpha+\omega_\beta)$ does not vanish for $\omega = 0$ and therefore provides an additional contribution Interestingly, our simulations finds that this contribution matches the value of *3/2* T_1, so that the total decoherence becomes T_2*= 0.5* T_1. The same result was obtained for the spin dynamics under the influence of hyperfine-mediated spin-phonon interaction.

Supplementary Figures 22,23 - Comparison with other VO-based systems

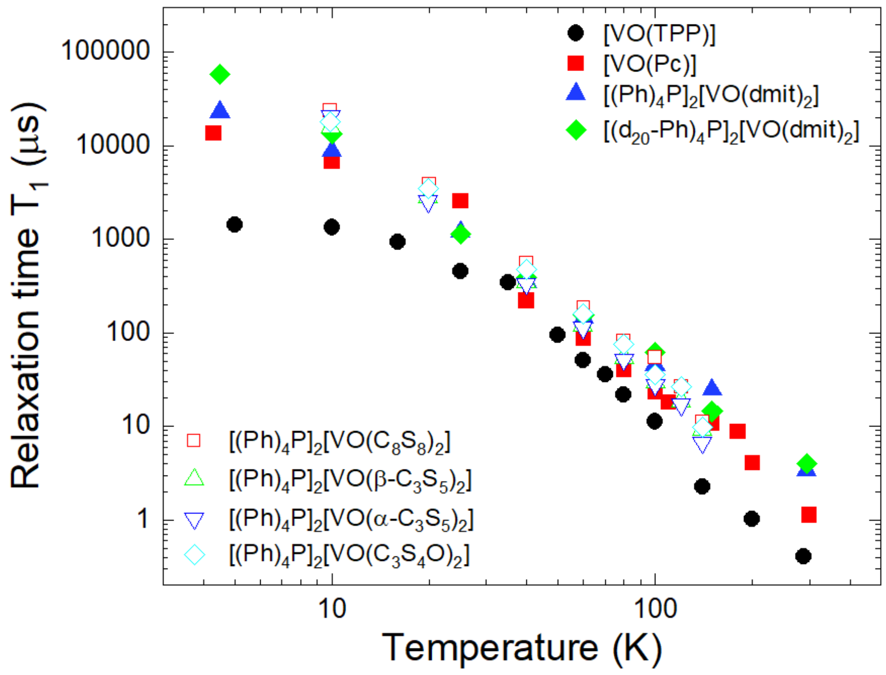

Supplementary Figure 22. Comparison of relaxation times: diluted crystals and frozen solutions. [VO(TPP)] experimental relaxation time T_1 (black circles) (Ref.(27) of the main text) compared with other VO-based systems in crystalline dispersions in their isostructural diamagnetic host as in Fig.7 of the main text: VOPc (red squares, 2% dilution, Ref.(53)), $[(Ph)_4P]_2[VO(dmit)_2]$ (blue triangles, 5% dilution, Ref.(54)) and $[(d_{20}\text{-}Ph)_4P]_2[VO(dmit)_2]$ (green diamonds, 5% dilution, Ref.(54)). Empty symbols are experimental relaxation times T_1 measured on frozen solutions of compounds reported in Ref.(14) of the main text: $[(Ph)_4P]_2[VO(C_8S_8)_2]$ (red squares), $[(Ph)_4P]_2[VO(\beta\text{-}C_3S_5)_2]$ (green triangles), $[(Ph)_4P]_2[VO(\alpha\text{-}C_3S_5)_2]$ (blue inverse triangles), $[(Ph)_4P]_2[VO(C_3S_4O)_2]$ (cyan diamonds) (error bars are within the size of the symbols).

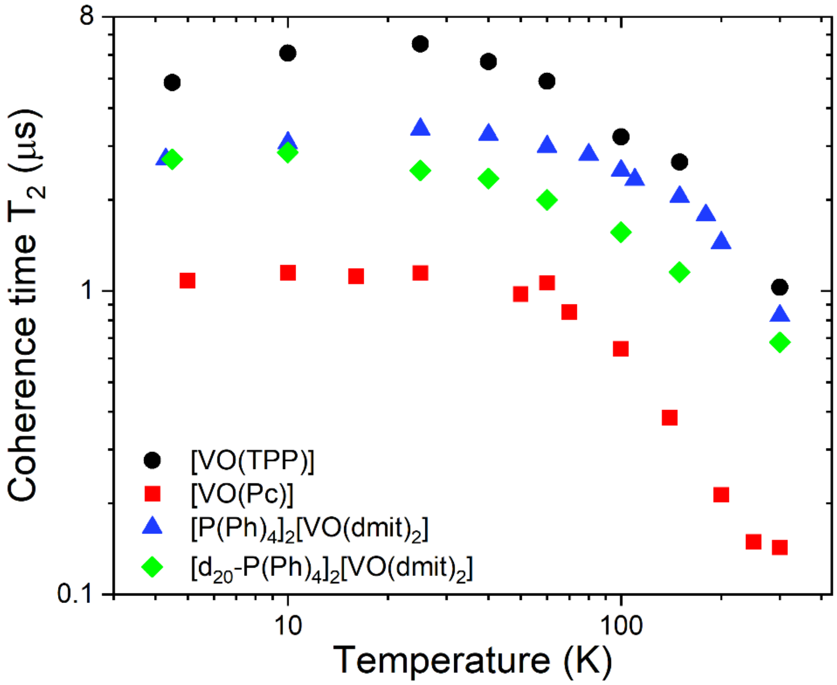

Supplementary Figure 23. Comparison of decoherence times. [VO(TPP)] experimental decoherence time T_2 (black circles) (Ref.(27) of the main text) compared with other VO-based systems: VOPc (Pc = phthalocyanine, red squares) (Ref.(53) of the main text), $[(Ph)_4P]_2[VO(dmit)_2]$ (dmit = 1,3-dithiole-2-thione-4,5-dithiolate, blue triangles) (Ref.(54) of the main text) and its analogue compound with a deuterated cation, $[(d_{20}\text{-}Ph)_4P]_2[VO(dmit)_2]$ (green diamonds) (Ref.(54) of the main text). All the samples were crystalline dispersions in their isostructural diamagnetic host: [TiO(TPP)] (2% dilution), TiOPc (0.1% dilution), $[(Ph)_4P]_2[MoO(dmit)_2]$ (5% dilution) and $[(d_{20}\text{-}Ph)_4P]_2[MoO(dmit)_2]$ (5% dilution) (error bars are within the size of the symbols).
