# Supplementary material for: The critical role of ultra-low-energy vibrations in the relaxation dynamics of molecular qubits
Source: Nat Commun. 2023 Mar 24;14:1653. doi: 10.1038/s41467-023-36852-y (PMC10039010; doi:10.1038/s41467-023-36852-y)
Supplement: Supplementary file 3 — Description of Additional Supplementary Files [file 41467_2023_36852_MOESM3_ESM.docx]

**Description of Additional Supplementary Files**

**File Name: Supplementary Movie 1
Description:** Molecular distortions associated to the first phonon mode at the Γ-point for a [VO(TPP)] single crystal.

**File Name: Supplementary Movie 2
Description:** Molecular distortions associated to the first vibrational mode of a [VO(TPP)] isolated molecule (gasphase calculations).
