# Supplementary figures and images for: The critical role of ultra-low-energy vibrations in the relaxation dynamics of molecular qubits
Source: Nat Commun. 2023 Mar 24;14:1653. doi: 10.1038/s41467-023-36852-y (PMC10039010; doi:10.1038/s41467-023-36852-y)

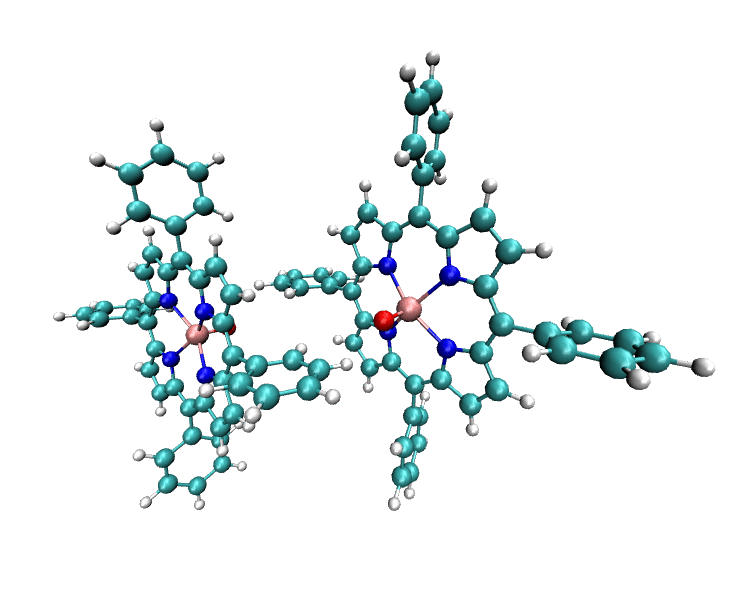

Supplement: Supplementary file 4 — Supplementary Movie 1 [file 41467_2023_36852_MOESM4_ESM.gif]

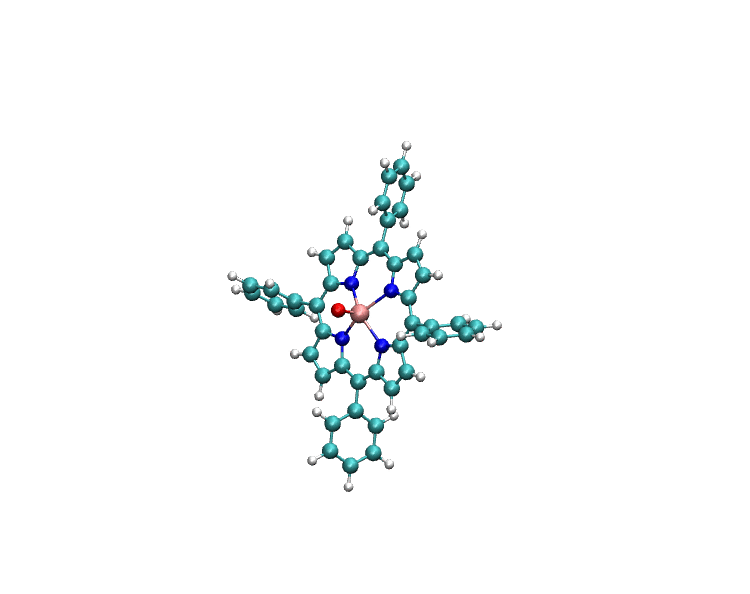

Supplement: Supplementary file 5 — Supplementary Movie 2 [file 41467_2023_36852_MOESM5_ESM.gif]
